# Response of grafting tobacco to low potassium stress

**DOI:** 10.1186/s12870-020-02481-6

**Published:** 2020-06-22

**Authors:** Wei Hu, Qing Di, Jie Zhang, Jia Liu, Xiaojun Shi

**Affiliations:** 1 College of Resources and Environment, Southwest University, Chongqing, 400716 China; 2 Vegetable and Flower Institute of Chongqing Academy of Agricultural Sciences, Chongqing, 401329 China; 3 Nanchang Institute of Technology, Nanchang, 330099 China; 4 Soil and Fertilizer & Resources and Environment Institute, Jiangxi Academy of Agricultural Sciences, Nanchang, 330200 China

**Keywords:** Grafting, Tobacco, Potassium stress, K^+^ channel current, Net K^+^ flux

## Abstract

**Background:**

In the previous study, we investigated the alleviation effect of grafting on potassium uptake in roots and tobacco growth inhibition under low potassium stress. However, the effect of grafting on the low potassium stress perception and coping mechanism of tobacco at the whole plant level is not clear now. In order to clearly understand the impact of grafting on potassium deficit responding mechanism in tobacco, a mutual grafting experiment has been conducted in two varieties of tobacco (‘Wufeng No.2’ and ‘Yunyan 87’) in different K supply level (5 mmol L^− 1^ and 0.5 mmol L^− 1^ K).

**Results:**

The results show that compared with the self-rooted seedlings, grafting significantly increased the potassium content of the whole plant of Yunyan 87 (97.57 and 189.74% under normal potassium and low potassium conditions, respectively), and the increase in shoots was greater. The data of whole plant K content distribution and tobacco hypocotyls net K^+^ flux demonstrates that potassium stress makes plants more inclined to maintain K^+^ in the shoot rather than root. In addition, when K deficiency occurs, grafting could reduce the time required for downward net K^+^ flux in tobacco hypocotyl to decrease to stable levels. The results of net K^+^ flux in the roots indicated that K channel proteins and transporters play different roles in two rootstocks in terms of potassium tolerance. Transcription level analysis suggested that the increased circulating efficiency of K^+^ between the shoots and roots in tobacco constitutes one means to low potassium stress adaptation.

**Conclusions:**

Grafting can activate more K^+^ channels in tobacco ‘Yunyan 87’, this means a more active K^+^ cycle, higher potassium content in shoot and faster response to low potassium stress signals in grafting tobacco. In addition, grafting can also change the K^+^ absorption mode of tobacco root from being dominated by HATS to being jointly responsible by HATS and LATS, greatly improving the ability of K^+^ transmembrane transportation on root surface under low potassium stress. These are undoubtedly the reasons why grafting tobacco performs better in coping with low potassium stress.

## Background

As a macronutrient for the growth of flue-cured tobacco (*Nicotiana tabacum*), potassium (K) is also an important limiting factor for high quality flue-cured tobacco in China. The formation of nucleic acids proteins, carbohydrates and the processes of photosynthesis, enzyme activation and osmoregulation are all associated with K [1, 2]. A common sense in the cigarette industry is that the importance of tobacco leaves K content in the quality evaluation system cannot be underestimated. Tobacco plants growing under conditions that allow for a sufficiently high accumulation of K are associated with a product with improved aromatic taste, flammability, and processability of the leaves [3]. But, for most tobacco planting regions in China, the low available K content in the tobacco planting soil makes K levels in tobacco leaves unable to meet the global standards for high-quality tobacco. According to a recent soil survey, 63.1% of tobacco planting soil in China is below the critical available K content level of 150 mg/kg. Of this soil, 19.6% is extremely K deficient, and the average available K content is only 57.5 mg/kg for this region. The remaining 43.5% constitutes K-deficient soil [4]. Improving the uptake and utilization efficiency of K in tobacco has thus become an important issue in tobacco farming.

K is highly mobile in plants and can be transported from the root to the shoot. This function plays a significance part in the electrical balance and energy conservation of plants. The K channel genes *SKOR* and *AKT2* determine the redistribution of K^+^ in plants. The former is abundantly expressed in the pericycle and xylem parenchyma cells in the root system, which mediate the transport of K^+^ to the shoots [5], while the latter is abundantly expressed in the phloem vascular system of the roots and leaves, which dominates the loading and unloading of K^+^ in the phloem [6]. When plants perceive external K deficit signals, cells produce both short-term and long-term response patterns: for short-term responses, *AKT1* expression is up-regulated, and K levels in the cytoplasm are maintained by releasing K^+^ in the vacuoles. For the long-term response, when the stress period lasts for several days or even weeks, the K^+^ concentration in the cytoplasm is decreased, the metabolic processes of the plant cells are affected, the activity of H^+^-PPase dependent on K^+^ activation is inhibited, and the pyruvate content is decreased [7]. Apart from this, cell elongation in the root elongation zone of plants requires cell swells produced by K^+^. If the K concentration is low, the root morphology of the plant will change. The growth of the main root is inhibited, and the growth of the root hair becomes strong. This adaptation to the environment is determined by the combination of NH_4_^+^ and growth hormones [8]. Functional complementation experiments of a mutant confirmed the involvement of ethylene in low-K stress signal transduction as well as its close regulation of root morphology [9]. Studies on the *Arabidopsis* auxin synthesis gene deletion mutants *aux1*, *axr1*, and *axr2* indicate that auxin is also a component of the response mechanism of the root morphology to K stress [10].

Grafting has been widely used for the improvement of crop quality, growth and yield; to alter varieties; to increase environment stress resistance; and to optimize cross-pollination [11–15]. Beyond that, grafting also has a significant impact on improving plant nutrient uptake [16–18]. However, few data in literature as regards the impacts of grafting on the tolerance of tobacco to K starvation stress. In the previous study [19], we investigated the alleviation effect of grafting on potassium uptake in roots and tobacco growth inhibition under low potassium stress. However, the effect of grafting on the low potassium stress perception and coping mechanism of tobacco at the whole plant level is not clear. This article aims at this goal, through the study of the genes related to potassium transport in phloem and xylem, the net K^+^ flux at the hypocotyl, the intensity of the inward current in K^+^ channel, the distribution mode of potassium content in the shoot and root of the tobacco plant to analyzes the adaptation mechanism of different genotypes of tobacco under low potassium stress and the influence of grafting on them.

## Results

### K distribution between the shoots and roots

**Fig. 1:**
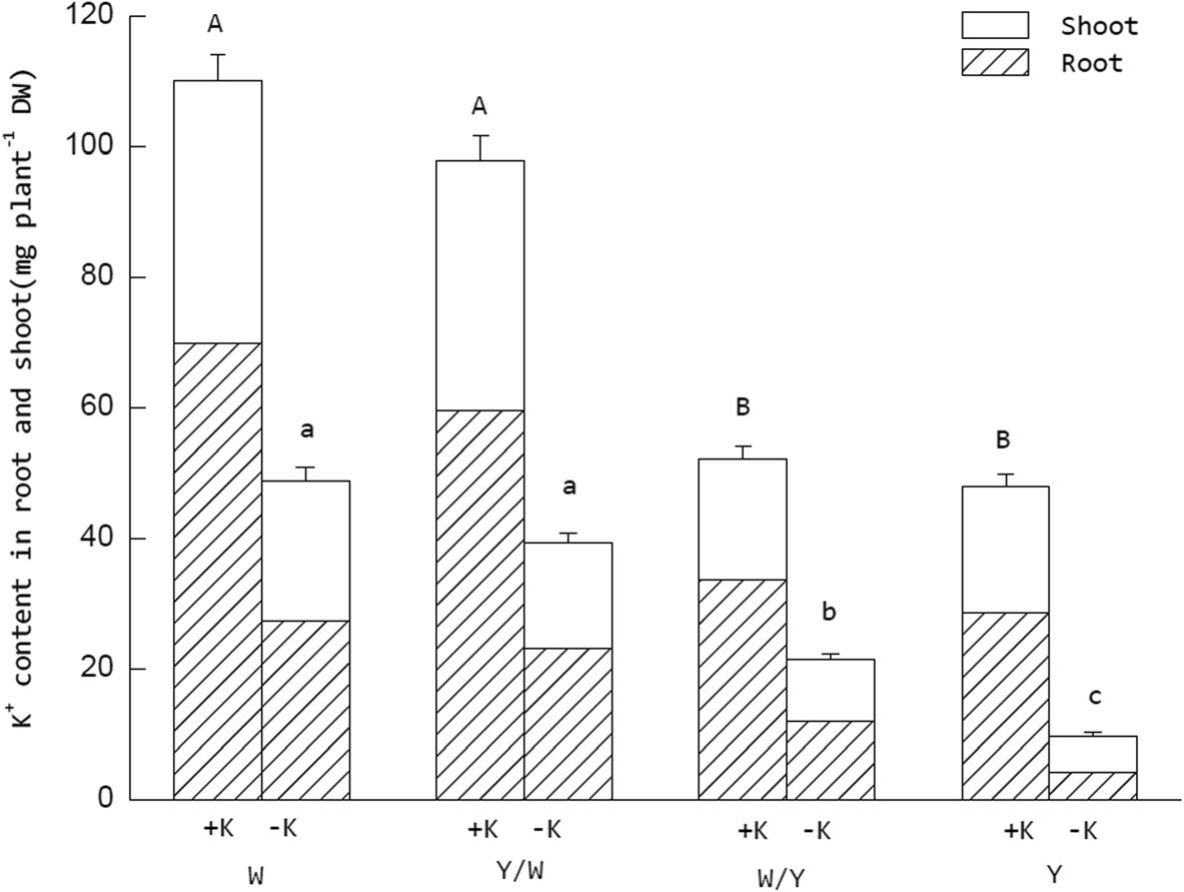
Effect of grafting combination and K^+^ supply level on K^+^ distribution in tobacco plants. The tobacco graft combinations included the nongrafted tobacco W (Wufeng No.2) and Y (Yunyan 87) and grafted tobacco Y/W (Y grafted onto W) and W/Y (W grafted onto Y). Different uppercase letters denote significant differences (*P* < 0.05) under normal K levels (5 mmol L^− 1^) in potassium content of whole plant, and lowercase letters indicate significant differences (*P* < 0.05) under starvation (0.5 mmol L^− 1^)

K^+^ absorption and distribution were both affected by the grafting treatment (Fig. 1). K stress significantly decreased the whole-plant K^+^ uptake capacity. However, the whole-plant K content performances of the different graft combinations differed. Under the +K treatments, whole K content in the treatments using K-efficient genotype tobacco ‘Wufeng No.2’ (W) as rootstock increased by 97.6% compared with The common cultivar ‘Yunyan 87’ (Y). In the –K treatments, this raised to 137.76%. In addition, differs from the data of the +K treatments, whole K content in W/Y significant higher than Y under low K supply. This demonstrates that W tobacco as the scion could improve the K^+^ absorption of genotype Y tobacco under K^+^ deficit. Under the –K treatments, the average K content percentage in the shoots accounted for 44.3% of the total plant, which was 7.3% higher than that in +K treatments. Furthermore, the tendency of the K in the tobacco plants to tilt toward the shoots under K stress was more pronounced in the W and Y/W grafting combinations.

### K^+^ channel inward current in root cells

**Fig. 2:**
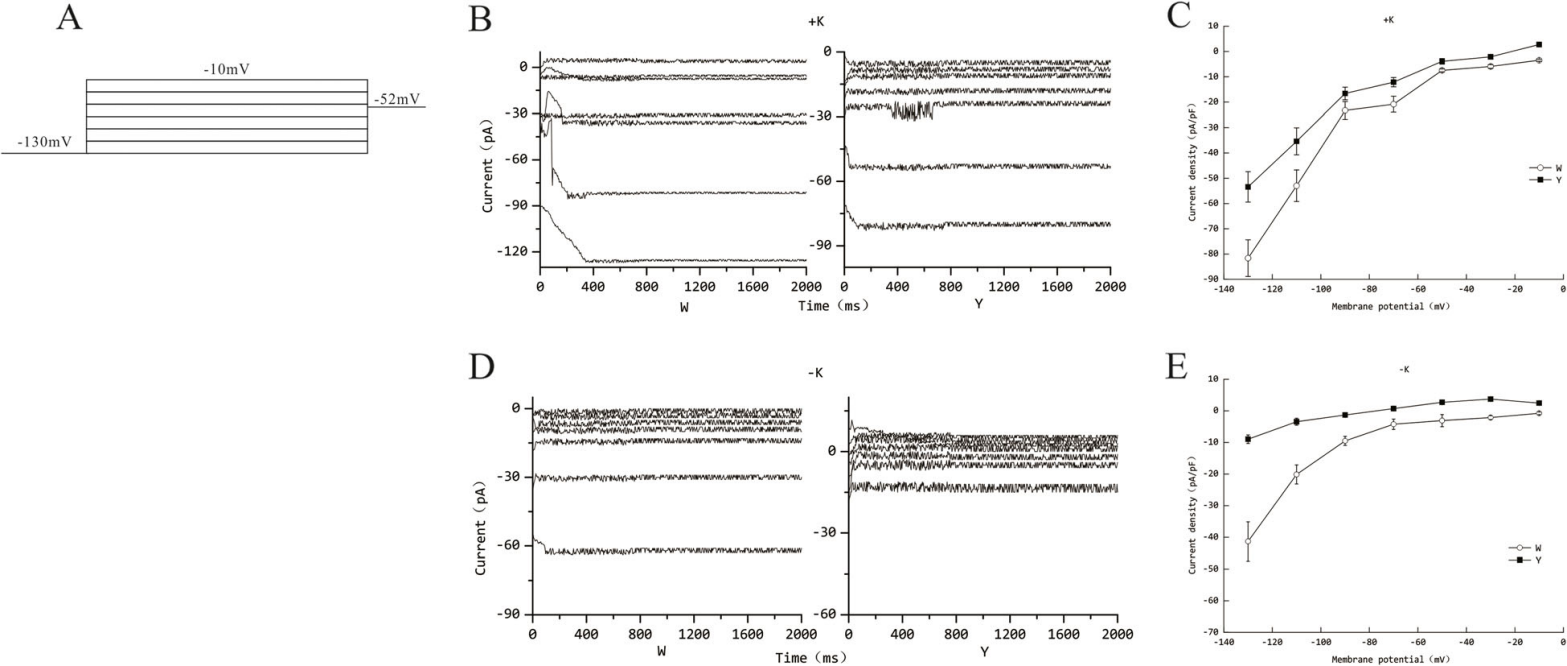
The inward current of K^+^ channel in flue-cured tobacco root cells under different potassium levels. **a**. The currents were recorded at the membrane potentials from − 130 to -10 mV (in 20 mV steps) with the holding potential of − 52 mV; **b**. The inward current of K^+^ channel with normal potassium supply; **c**. Current amplitude reflected by I-V curve with normal potassium supply; **d**. The inward current of K^+^ channel with low potassium stress; **e**. Current amplitude reflected by I-V curve with low potassium stress

The potential difference generated by K^+^ transmembrane transport in the protoplasts of root cells is the current intensity of K^+^ channel, which could reflect the transport capacity of K^+^ mediated by channel proteins. The inward currents in the K^+^ channel could be recorded by patch-clamp to evaluate the effects of different treatments on the K^+^ channel transport function in root cells. As shown in Fig. 2, with the normal potassium supply, the maximum value of the inward current in K^+^ channel of root cells of W genotype was − 127.83pA, while that in Y was − 82.57pA. With the low potassium stress, the maximum value of the K^+^ channel inward current of root cells in the two genotypes decreased significantly, with a decrease of 52.33% in W and 85.15% in Y. From the analysis of the I-V curve, it can be known that with the normal potassium supply, the current density of W genotype was 81.33pA/pF at − 130 mV voltage and that of Y was 53.44 pA/pF, which was reducing 34.28%. Under the condition of low potassium, the decrease rate reached 78.5%. Moreover, the I-V curve of genotype Y was always above that of W regardless of the potassium level. This result first indicates that the transmembrane transport efficiency of K^+^ channel was weakened under low potassium stress; Secondly, regardless of the potassium level, the K^+^ channel inward current intensity of genotype W was always stronger than that of Y, which was one of the difference sources in potassium absorption and transport capacity between the two flue-cured tobacco genotypes.

### The response of net K^+^ flux in tobacco hypocotyls under K stress

**Fig. 3:**
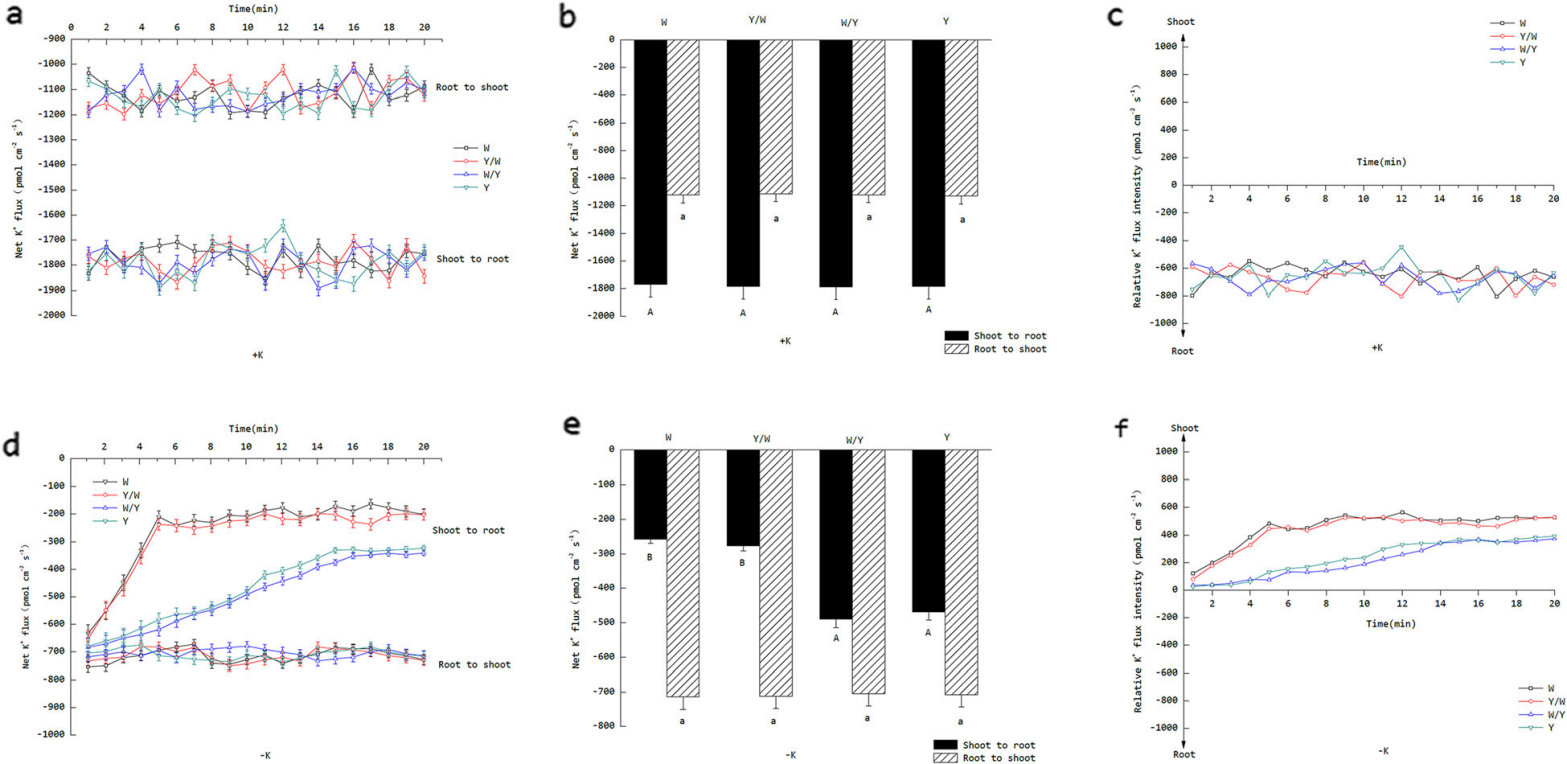
Effects of grafting on the net K^+^ flux in tobacco hypocotyls. The tobacco graft combinations included the nongrafted tobacco W (Wufeng No.2) and Y (Yunyan 87) and grafted tobacco Y/W (Y grafted onto W) and W/Y (W grafted onto Y). **a** The net K^+^ flux dynamics of the hypocotyls under normal K levels (5 mmol L^− 1^) over 20 min. The net K^+^ flux at transverse sections of the shoot constituted the downward ion current of the phloem, which was defined as “Shoot to root,” while the net K^+^ flux at transverse sections of the root constituted the upward ion current of the xylem, which was defined as “Root to shoot.” For all ion flux measurements, the sign convention is “influx positive.” (**b**) The mean net K^+^ flux of the hypocotyls under normal K levels (5 mmol L^− 1^) over 20 min. Different uppercase and lowercase letters denote significant differences (*P* < 0.05) between each grafting combination. (**c**) The relative K^+^ flux under normal K levels (5 mmol L^− 1^). The relative K^+^ flux is calculated by subtracting the downward ion current from the upward ion current. The numerical quadrant represents the direction of the relative flux of K^+^. (**d**) The net K^+^ flux dynamics of the hypocotyls under K starvation (0.5 mmol L^− 1^) over 20 min. (**e**) The mean net K^+^ flux of the hypocotyls under K starvation (0.5 mmol L^− 1^) over 20 min. (**f**) The relative K^+^ flux under K starvation (0.5 mmol L^− 1^)

The experimental data show that when the K concentration has changed in the external environment, the contrast of the upward and downward net K^+^ flux at the transverse sections of the tobacco hypocotyls was also altered. In the +K treatments, the mean net K^+^ shoot-to-root flux in the transverse section of the hypocotyls was 1782.72 pmol cm^− 2^ s^− 1^, which was 1.59 times higher than that of the root to shoot (Fig. 3a). Additionally, there was no significant difference in individual upward or downward net K^+^ flux between each grafting combination (Fig. 3b). In order to clarify the relative direction of K^+^ movement in long-distance transportation under different K levels, the value obtained by subtracting the downward net K^+^ flux from upward net K^+^ flux has been defined as the relative K^+^ flux intensity. The relative K^+^ flux intensity in each treatment was negative during the entire measurement period under the +K treatments, which suggests that K^+^ in the tobacco plants tends to move from the shoot to the root when the K supply is sufficient (Fig. 3c). In the –K treatments, the K^+^ flux from the root to shoot of each grafting combination was stable throughout the entire measurement period, and the difference between each grafting combination was not significant. However, the rate of K^+^ flux from the shoot to root in the treatments using W as the rootstock decreased rapidly after the start of the test and gradually stabilized after 5 min. As for the treatments using Y as the rootstock, the rate of K^+^ flux was stable after 16 min of decline (Fig. 3d). The mean net K^+^ efflux of the shoot to root in the transverse section of the hypocotyls was 373.45 pmol cm^− 2^ s^− 1^, which was 52.3% of that of the root to shoot. At the same time, the net K^+^ flux from the shoot to root in the treatments using W as the rootstock was significantly lower than the treatments using Y as the rootstock (Fig. 3e). Contrary to the +K treatments, the relative K^+^ flux intensity in each treatment was positive during the entire measurement period under the –K treatments, which indicates that K^+^ in the tobacco plants tended to move from the root to the shoot when the K supply was insufficient (Fig. 3f).

### The effects of K deficit on the acquisition of K^+^ by the tobacco roots

**Fig. 4:**
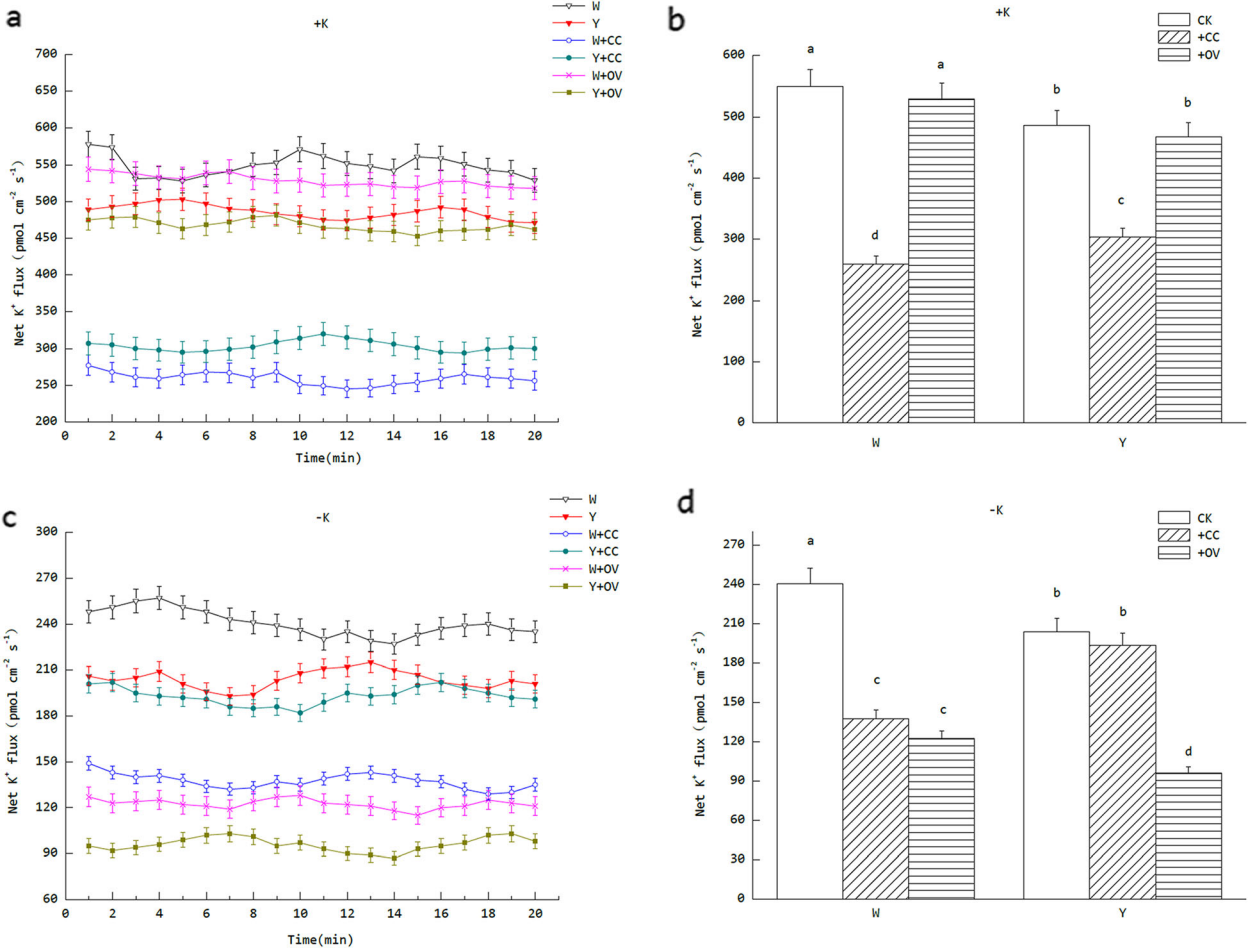
Net K^+^ flux in the primary root meristem under different concentrations of K, with or without inhibitors (W: tobacco Wufeng No.2; Y: tobacco Yunyan 87; CC: cesium chloride, a potassium ion channel inhibitor; OV: orthovanadate, a plasma membrane H^+^-ATPase inhibitor). **a** The net K^+^ flux dynamics of the root meristem under normal K levels (5 mmol L^− 1^). For all ion flux measurements, the sign convention is “influx positive.” (**b**) The mean net K^+^ flux of the root meristem under normal K levels (5 mmol L^− 1^). Different lowercase letters denote significant differences (*P* < 0.05). (**c**) The net K^+^ flux dynamic of the root meristem under K starvation (0.5 mmol L^− 1^). **d** The mean net K^+^ flux of the root meristem under K starvation (0.5 mmol L^− 1^). Different lowercase letters denote significant differences (*P* < 0.05)

According to the data analysis of K^+^ flux in the tobacco roots, significant differences in the strategies of the tobacco varieties W and Y in tolerating low K stress in the environment were observed. In the +K treatments, the mean net K^+^ influx of W in the root meristem was 549.05 pmol cm^− 2^ s^− 1^, which was 1.13-times higher than that of Y. When the two tobacco rootstocks were pretreated with cesium chloride (a potassium ion channel inhibitor), the net K^+^ influx in both treatments was significantly lower than the rootstocks without the inhibitor. In addition to this, the decline of mean net K^+^ influx in W roots was 52.6%, but only 37.5% in Y. However, when the pretreatment agent was replaced with orthovanadate (a plasma membrane H^+^-ATPase inhibitor), there was no significant effect on the net K^+^ influx in the roots of the two treatments compared with the original rootstocks (Fig. 3a and b). In the –K treatments, the mean net K^+^ influx of W in the root meristem was still significantly higher than Y. However, when the specific inhibitors were involved in the trial, the situation differed from that of the +K treatments. Cesium chloride significantly reduced the K^+^ flux in the W root meristem, whereas there was no significant effect on Y (Fig. 4c and d). However, orthovanadate had a significant effect on net K^+^ flux in both the tobacco rootstocks, with Y exhibiting a greater decline (the decrease was 15.4% more than W).

### Effects of grafting on the expression of genes related to K absorption and transport

**Fig. 5:**
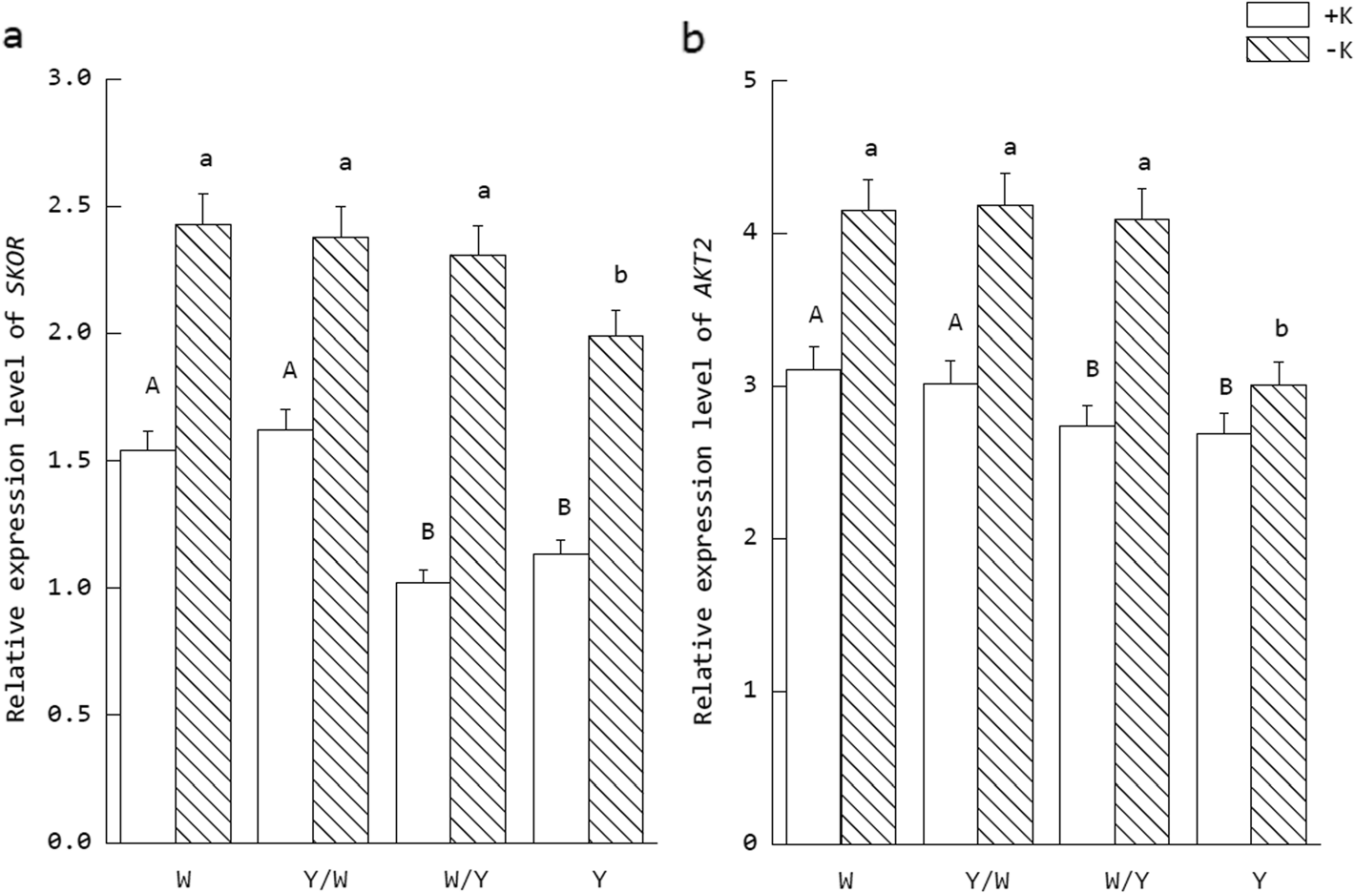
Relative expression levels of genes (a. *SKOR*, b. *AKT2*) related to K absorption and transport in tobacco with two K supply conditions. The tobacco graft combinations included the nongrafted tobacco W (Wufeng No.2) and Y (Yunyan 87) and grafted tobacco Y/W (Y grafted onto W) and W/Y (W grafted onto Y). Different uppercase letters denote significant differences (*P* < 0.05) under normal K levels (5 mmol L^− 1^), and lowercase letters indicate significant differences (*P* < 0.05) under K starvation (0.5 mmol L^− 1^)

The qRT-PCR results indicated that the transcription level of *SKOR* and *AKT2* in the tobacco plants had been significantly increased by K deficit. As reveal in Fig. 5, for the different graft combinations, the transcription levels in the groups with W as the rootstock were significantly higher than those groups with Y as the rootstock under normal K supply levels. A similar situation was observed under K starvation. However, the expression level of *SKOR* and *AKT2* in the W/Y treatments was significantly higher than Y when K deficiency and it was not significantly lower than the W and Y/W grafting treatments.

## Discussion

### K starvation changes the distribution trend of K^+^ in tobacco

Different genotypes in the same crop variety sometimes differ greatly in nutrient uptake and transformation. Grafting can effectively utilize this feature to combine nutrient-efficient rootstocks with target scions to improve crop quality and yield [20–23]. In tobacco, the uptake of multiple nutrients can be influence by grafting [24, 25]. In our study, the W tobacco was not only superior to the Y in terms of whole K content, but its performance was also enhanced when W was used as the rootstock. Regardless of whether there was normal K supply or K starvation, the K content of the plants with the W genotype tobacco rootstock was significantly higher than the plants with the Y genotype. Combined with our previous research [19], grafting not only improved tobacco growth, but also increased the whole-plant K content of the Y genotype tobacco compared with the nongrafted original variety. Another interesting observation in the experiment was that the distribution of K^+^ in the tobacco plant changed under K starvation. Regardless of the K supply level, the K content in the roots is typically the highest in the entire plant. However, the tobacco plants were more willing to retain K^+^ in the shoots than transport it to the roots under K-deficient conditions, which constitutes one of the mechanisms that tobacco plants use to tolerate K stress. Furthermore, this tendency was more pronounced in the W and Y/W grafting combinations. When the demand for nutrients in the plant shoots increases, the nutrient concentration through the phloem to the root system decreases, and as a feedback signal, the absorption rate of the ions by the roots is promoted. Similarly, when the demand for nutrients decreases in the shoots, the concentration of nutrients circulating in the phloem increases and inhibits the absorption of the corresponding ions by the roots [26, 27].

### The difference between two genotype flue-cured tobacco of K^+^ channel current in root cells

The Shaker family of the K^+^ channel is currently the most studied channel protein family. It is generally believed that S4 in its six transmembrane domains (S1-S6) is the transmembrane voltage signal receptor [5], which can stimulate the opening of the channel. While the ring structure between S5 and S6 is responsible for the transmembrane crossing of ions due to its high degree of conservation. The Shaker family can be divided into three types: inward rectifying, outward rectifying and weak inward rectifying, depending on voltage dependence and K^+^ transportation direction. The first one is involved in this experiment [28]. The driving force of K^+^, which is transferred in the form of passive diffusion in the K^+^ channel, is generated by the potential difference between the membrane and the proton pump out of the membrane. Changes in the external environment, especially abiotic stress factors, can cause stress responses in plants. This not only increases the level of calmodulin, but also accelerates the metabolism of polyamines and reactive oxygen species (ROS), while inhibiting the inward current of K^+^ channels [29]. The experimental data show that low potassium stress would decrease the inward current density of K^+^ channel, but the decline of different genotypes of flue-cured tobacco was not consistent. The inward current of K^+^ channel of Y genotype flue-cured tobacco is more affected in the face of low potassium stress. However, the inward current density of K^+^ channel in W genotype flue-cured tobacco roots was higher than that in Y regardless of potassium levels. *NKT1* is an inwardly rectifying K^+^ channel gene that mediates K^+^ uptake in Shaker-like family expressed in tobacco cells [30]. In our previous studies, it was found that low potassium levels can induce down-regulation expression of this gene [19]. This corresponds to the results of this experiment. By reducing unnecessary consumption, activating more efficient potassium transport pathways (such as potassium transport proteins) may be one of the strategies of potassium efficient flue-cured tobacco to deal with potassium deficit.

### Net K^+^ flux in the transverse section of the hypocotyls and root meristem

K^+^ from the root surface is transversely transported to parenchyma cells and transported from the root to shoot through xylem loading [31], while the phloem circulates K^+^ back to the roots. K^+^ transport in phloem and xylem is adjusted by the K concentration in each tissue of plant [32]. The experimental data show that the net K^+^ flux transported through the phloem to the root was higher than the net K^+^ flux transported to the shoot by the xylem under +K treatments. In the case of the –K treatments, the situation was reversed. The net K^+^ flux of the “root to shoot” was higher than the “shoot to root.” This phenomenon confirms the above conclusions regarding the distribution pattern of K^+^ under different K levels. Under K deficit, the time required for the downward net K^+^ flux to decrease to a stable state was the same when the rootstocks were the same, and the scions were different. But when the rootstocks differed and the scions were the same, the time required for downward net K^+^ flux to decrease to stable levels was inconsistent (the treatments using W as a rootstock stabilized at 5 min, whereas those using Y required 16 min). This indicates that the sensory mechanism of the K concentration change of the tobacco plant in the external environment is dominated by the rootstocks rather than the scion. This sensory mechanism is mainly derived from the changes in potential on the cell membrane, which is unusually sensitive and is typically observed within several minutes of a decrease in ambient K levels [33, 34]. Studies have shown that there are two major receptors that produce the above potential changes, including the K^+^ channel gene of the Shaker family (mediated by the *AKT1*) and H^+^-PPase [7]. In addition, the time required for downward net K^+^ flux to decrease to stable levels in treatments using W as the rootstock was less than that in those using Y. This demonstrates that W-genotype tobacco can alter the distribution pattern of K in plants more rapidly in the presence of K stress and can maintain normal K levels in the shoot by reducing K circulation in the roots. The ability of the W genotype tobacco to rapidly respond to K stress was transmitted to the Y tobacco through grafting, thus allowing it to respond better to K deficiency.

High-affinity transport system (HATS) and low-affinity transport system (LATS) are the two way for plants absorption of K^+^ from interface of root and soil [35]. It is evident from the experimental data that the absorption rate of K^+^ in the root meristem of the W genotype tobacco was significantly higher than that of Y in the absence of inhibitors. Experiments conducted with specific inhibitors indicate that the dominant K^+^ absorption pattern of the two tobacco rootstocks under normal K supply levels was a LATS that depended on K ion channels. The K^+^ absorption method of the W rootstock was operated by LATS and HATS concurrently when K deficit. The HATS transportation volume was larger but the difference with LATS was not significant, which indicates that the status of both K^+^ absorption systems was equally important under K stress. In addition, the K^+^ absorption mode in the root meristem of the Y rootstock under K deficiency was mainly HATS, whereas LATS was inhibited. The change in membrane potential can be used as a plant response signal to K deficiency. When the concentration of exogenous K^+^ was low, the plasma membrane was hyperpolarized to activate the K channel. Conversely, when the concentration of exogenous K^+^ was high, the plasma membrane was depolarized. The results of this study indicate that K channel proteins and transporters in W genotypes play the same important role in coping with K stress, while only the transporters play a leading role in Y. This is one of the reasons for the difference in K^+^ absorption between two tobacco varieties. Similar findings were also found in salt-tolerant pumpkin rootstocks [36].

### Analysis of gene expression related to the K transport pathway

The K^+^ channel family [5], high affinity transporter family [37], co-transporter family [38], reverse transporter family, and proton pump genes for energy supply [39] were involved in regulated the process of uptake and transport of K in tobacco. In the K^+^ channel gene family, *SKOR* is responsible for the outward flow of K^+^ from xylem, and *AKT2* is involved in the loading and unloading of K^+^ in phloem. The former is responsible for the outward rectifying transport of K^+^, while the latter is responsible for the weak inward rectifying transport [30]. Studies have shown that *AKT2* deletion mutation will cause a decrease in the cell membrane potential of phloem cells and affect the concentration of sucrose in the tissue [40]. In terms of transcriptional regulation, *AKT2* was not only affected by photophosphorylation, but also induced by nutrient stress. The lack of potassium could lead to the up-regulated expression of *AKT2* in wheat root cells and the activation of K^+^ channel. The effect of low potassium level on *SKOR* and *AKT2* was opposite in Arabidopsis [41, 42]. In this experiment, the transcriptional expression was affected by both potassium levels and grafting. The up-regulated expression of *SKOR* and *AKT2* in low K stress suggests tobacco could improve the transport efficiency of K^+^ by activating more K^+^ channels in shoot to cope with low potassium stress. Notably, the behavior of the W/Y grafting combination under the -k treatment indicates that this regulation can be transmitted between the root stock and scion by the signal substance.

## Conclusion

Grafting with potassium efficient genotype tobacco “Wufeng No.2” as rootstock can activate more K^+^ channels in the shoot of “Yunyan 87”. This means a more active K^+^ cycle, higher potassium content in shoot and faster response to low potassium stress signals in grafting tobacco. In addition, grafting can also change the K^+^ absorption mode of tobacco root from being dominated by HATS to being jointly responsible by HATS and LATS, greatly improving the ability of K^+^ transmembrane transportation on root surface under low potassium stress. These are undoubtedly the reasons why grafting tobacco performs better in coping with low potassium stress. However, these advantages of grafted tobacco are based on the rapid response of low potassium stress signals. Therefore, to provide insight into the signaling pathway of grafted tobacco in response to low potassium stress, the co-expression analysis of differentially expressed genes and differentially accumulated metabolites in grafted tobacco will be our next work need to do.

## Methods

### Plant materials and treatments

The experiments were conducted in the greenhouse of the Chongqing Academy of Agricultural Sciences, Chongqing, China (29°36′N, 106°29′E). The common cultivar ‘Yunyan 87’ (*N. tabacum*, Yunnan Tobacco Research Institute, China) and K-efficient genotype tobacco ‘Wufeng No.2’ (*Nicotiana tabacum*, Yichang Tobacco Company of Hubei Province, China) were used in the present research. In order to ensure the success rate of grafting, the seeds of rootstock should be planted 7 days before the scion. The ‘split grafting’ was selected as the grafting method when six to eight true leaves had appeared on the seedlings of the rootstock. When the new leaves had grew by grafted plants, transplanted the plants into hydroponic box, with 12 seedlings planted per container. The nutrient solution for hydroponics was formulated as previous study [19]. Air pumps were adopted to supply oxygen to each container through a hose for oxygen supply to the tobacco seedlings during experiment. The plants were grown at 22.5 °C under a 16-h light/8-h dark cycle using fluorescent lamps with an average photosynthetic photon flux density (PPFD) of 300 μmol m^− 2^ s^− 1^ in the greenhouse. The relative humidity ranged from 60 to 95%.

Two K levels were used for the experiments: +K, sufficient supply (5 mmol L^− 1^) and -K, deficit (0.5 mmol L^− 1^), using K_2_SO_4_ as the substance source to adjust different K levels. Besides that, four graft treaments [Wufeng No.2 (W), W/Y (W grafted onto Y), Yunyan 87 (Y), and Y/W (Y grafted onto W)] were used in experiment. Eight treatments were replicated 6 times with 12 plants in each replicate. The solutions were replaced every 4 d.

### K content determination in the tobacco

The tobacco plants were cut into two sections at the hypocotyl below the cotyledon. The stem above the cotyledon was defined as the “Shoot,” while the stem below the cotyledon was defined as the “Root.” A flame atomic absorption spectrometer was adopted for determining K content in the dried samples (Varian AA-220FS, Thermo Fisher Scientific, USA) as previously described [43].

### Root cell K^+^ channel inward current measurement

Fifteen days after transplanting, the fibrous roots of W and Y genotypes tobacco were collected and washed with deionized water, after then cut them into 5 mm segments. The treated sample was transferred to 1 ml of the enzymatic hydrolysate (1% cellulase, 0.15% pectinase, 0.8 mol L-1 mannitol, cell wash solution, pH 5.5–6.0), shaken for 2 h, 70 rpm, temperature 28 °C. The enzyme hydrolysates were then filtered with a 200-mesh filter and rinsed twice with cell washing solution under dark conditions. The filtrate was collected, centrifuged for 5 min at 100 rpm, removed the supernatant and repeated twice. The sediment was the protoplasm sample.

Select a suitable hard thin glass tube, and use a two-step method to draw the microelectrode on the drawing instrument (Narishige Japan) and polish it. The inner diameter of the tip is about 0.5-1 μm, and the intracellular fluid is injected into it [100mmo1 L^− 1^ potassium glutamate, 2 mmo1 L^− 1^ magnesium chloride, 0.1 mmo1 L^− 1^ calcium chloride, 10 mmo1 L^− 1^ 4-hydroxyethylpiperazine ethane sulfonic acid, 1.1 mmo1 L^− 1^ ethylene glycol double (2-aminoethyl ether) tetraacetic acid, 2 mmo1 L^− 1^ adenosine triphosphate, pH 7.2. Mannitol adjusts the osmolality to 800 mmo1 kg^− 1^]. The resistance of the electrode is 5–8 MΩ. Protoplasts were transferred to a sample pool containing 2 ml cell extracellular fluid (1 mmo1 L^− 1^ calcium chloride, 10mmo1 L^− 1^ potassium glutamate, 5mmo1 L^− 1^ 2-morpholine ethanesulfonic acid, 4mmo1 L^− 1^ magnesium chloride, pH 6.0. Mannitol adjusts the osmolality to 900 mmo1 kg^− 1^). After forming a high-resistance seal (resistance 1–5 GΩ) between the tip of the glass microelectrode and the plasma membrane, begin to apply a negative pressure to the inner cavity of the electrode. During stimulation, the voltage was depolarized from − 130 mV to − 10 mV, each stage was 20 mV, the duration was 2 s and the frequency was 0.2 Hz. Current signal and membrane capacitance were recorded by Axopatch-200B patch clamp amplifier, data acquisition card and Pclamp 6.0 software. The acquisition process was constant at room temperature (25 ± 1 °C). The channel current density was used to measure the whole cell current intensity (pA/pF, the ratio of current to cell capacitance) of each treatment, and the current amplitude was analyzed by I-V curve.

### Measurement of K^+^ fluxes in transverse sections of the tobacco hypocotyls

Six plants of each grafting combination in the +K treatment were chosen to measure net K^+^-flux with noninvasive micro-test technology (NMT system BIO-IM; Younger Corp., Amherst, MA, USA), ASET 2.0 (Sciencewares, Falmouth, MA, USA) and iFluxes 1.0 (YoungerUSA, LLC, Amherst, MA, USA) software [44, 45], as described previously [46]. Net K^+^ flux was calculated by Fick’s law of diffusion [47].

Half of the plants were treated with 0.5 mmol L^− 1^ K_2_SO_4_ for 2 h before measurement to observe the short-term reaction of each grafting combination under K stress. The tobacco plants were cut into two sections at the hypocotyl below the cotyledon and then fixed in measuring solution with belts. The transverse section of the hypocotyl was immediately incubated in the measuring solution [0.1 mmol L^− 1^ CaCl_2_ and 0.3 mmol L^− 1^ 2-ethanesulfonic acid (MES), pH 6] to equilibrate for 30 min. The equilibrated samples were then transferred to the measuring chamber filled with the solution containing either 0.5 mmol L^− 1^ K^+^ or 5 mmol L^− 1^ K^+^. The electrode was fixed at the center of the transverse section. Net K^+^ fluxes were measured under the experimental conditions for 20 min to decrease variability due to fluctuations. Each plant was measured once.

### Measurement of K^+^ fluxes in the root meristem

This test only involves the absorption of nutrients by the roots. Thus, only nongrafted tobacco W (Wufeng No.2) and Y (Yunyan 87) were adopted in this trial. The measuring site was the root meristem. In order to elucidate the possible effects of K^+^ channel and plasma membrane H^+^-ATPase compounds on K^+^ uptake, the net K^+^ fluxes at the root meristem were monitored after the application of orthovanadate, which is a specific inhibitor of PM H^+^-ATPases [48], and cesium chloride, which is a K channel blocker. The rest of the measurement methods were consistent with the measurement of K^+^ fluxes in transverse sections of the tobacco hypocotyls.

### Total RNA extraction and quantitative real-time (qRT) PCR

Total RNA was extracted from stems of ungrafted tobacco and grafted tobacco (*SKOR* and *AKT2*) with TRIzol reagent (Invitrogen, ThermoFisher, USA) according to the manufacturer’s instructions. 1 μg of total RNA in a 20-μL reaction system was used to synthesis first-strand cDNA in combination with oligo (dT)-18 as a primer and M-MuLV reverse transcriptase (TaRaKa, Japan). qRT-PCR (ABI 7900HT, Applied Biosystems, USA) was performed by a LightCycler480 SYBR Green I Master kit according to the protocols. Each sample analysis was repeated at least three times. The design of gene-specific primers was made by Primer premier5.0 software and all primers were summarized in Table 1. Each primer presented high specificity by the melting curve analysis. The PCR products were quantified by the 2^−ΔΔCt^ method [49].

**Table 1:** Primers used in the qRT-PCR analysis

Gene	Accession Number	Forward primer (5′-3′)	Reverse primer (5′-3′)
*SKOR*	NM_001326274	TCAGCCTTACACGGTTAGAGTTG	CACCGTAGAAAGCCGCACT
*AKT2*	NM_001325653	ACAAGACAATGCCACAATGCTC	AGGAGGAACAACATCGGTGT
Actin^a^	AB158612	AACAGTTTGGTTGGAGTTCTGG	CATGAAGATTAAAGGCGGAGTG

^a^Reference gene (Act) for qRT-PCR analysis

### Statistical analysis

All data in research are presented as the means of six replicates ± S.D. A two-factorial ANOVA was performed to determine the impacts of grafting on tobacco by SAS Version 9.3 (Statistical Analysis System Institute Inc., Cary, NC, USA). The significance differences between treatments was compared by Duncan’s multiple range test (*P* < 0.05).

## Data Availability

The datasets used and/or analysed during the current study available from the corresponding author on reasonable request.

## Abbreviations

*AKT2*: *Nicotiana tabacum* potassium channel AKT2/3-like
*SKOR*: *Nicotiana tabacum* Stelar K^+^ outward rectifier
qPCR: quantitative real-time PCR
